# Effects of an injectable long-acting formulation of ivermectin on *Onchocerca ochengi* in zebu cattle

**DOI:** 10.1051/parasite/2020036

**Published:** 2020-05-18

**Authors:** Michel Boussinesq, Peter Enyong, Patrick Chounna-Ndongmo, Abdel-Jelil Njouendou, Sébastien David Pion, Anthony Rech, Christophe Roberge, Georges Gaudriault, Samuel Wanji

**Affiliations:** 1 Recherches Translationnelles sur le VIH et les Maladies Infectieuses (TransVIHMI), UMI233 IRD-U1175 INSERM-Université de Montpellier BP 64501 34394 Montpellier Cedex 5 France; 2 Research Foundation for Tropical Diseases and the Environment PO Box 474 Buea Cameroon; 3 MedinCell S.A. 3 Rue des Frères Lumière 34830 Jacou France

**Keywords:** Onchocerciasis, *Onchocerca ochengi*, Ivermectin, Long-acting formulation, Microfilaricidal effect, Macrofilaricidal effect

## Abstract

The availability of a safe macrofilaricidal drug would help to accelerate onchocerciasis elimination. A trial was conducted in Cameroon to evaluate the effects of a subcutaneous injectable long-acting formulation of ivermectin (LAFI) on the microfilariae (mf) and adult stages of *Onchocerca ochengi*. Ten zebu cattle naturally infected with the parasite were injected subcutaneously with either 500 mg (group A, *N* = 4), or 1000 mg long-acting ivermectin (group B, *N* = 4) or the vehicle (group C, *N* = 2). Skin samples were collected from each animal before, and 6, 12, and 24 months after treatment to measure microfilarial densities (MFDs). Nodules excised before, and 6 and 12 months after treatment were examined histologically to assess the adult worms’ viability and reproductive status. Blood samples were collected at pre-determined time-points to obtain pharmacokinetic data. Before treatment, the average *O. ochengi* MFDs were similar in the three groups. Six months after treatment, all animals in groups A and B were free of skin mf, whereas those in group C still showed high MFDs (mean = 324.5 mf/g). Only one ivermectin-treated animal (belonging to group A) had skin mf 12 months after treatment (0.9 mf/g). At 24 months, another animal in group A showed skin mf (10.0 mf/g). The histologic examination of nodules at 6 and 12 months showed that LAFI was not macrofilaricidal but had a strong effect on embryogenesis. The new LAFI regimen might be an additional tool to accelerate the elimination of human onchocerciasis in specific settings.

## Abstract (fr)

La disponibilité d’un médicament macrofilaricide et sans danger permettrait d’accélérer l’élimination de l’onchocercose. Un essai a été mené au Cameroun pour évaluer les effets d’une formulation injectable en sous-cutané d’ivermectine à activité prolongée (FIAP) sur les microfilaires (mf) et les stades adultes d’*Onchocerca ochengi*. Dix vaches zébu infectées naturellement par le parasite ont reçu une injection sous-cutanée de 500 mg (groupe A, *N* = 4) ou de 1000 mg d’ivermectine à activité prolongée (groupe B, *N* = 4) ou le véhicule (groupe C, *N* = 2). Des échantillons de peau ont été collectés de chaque animal avant, puis 6, 12 et 24 mois après traitement pour mesurer les densités microfilariennes (DMF). Des nodules prélevés avant et 6 et 12 mois après traitement ont été examinés histologiquement pour évaluer la viabilité et le statut reproductif des vers adultes. Des échantillons de sang ont été prélevés pour obtenir des données de pharmacocinétique. Avant traitement, les DMF à *O. ochengi* étaient similaires dans les 3 groupes. Six mois après traitement, aucun des animaux des groupes A et B ne présentait de mf dermiques, alors que ceux du groupe C présentaient encore des DMF élevées (moyenne : 324,5 mf/g). Parmi les animaux traités par ivermectine, un seul (du groupe A) avait des mf dermiques 12 mois après traitement (0,9 mf/g). A 24 mois, un autre animal du groupe A avait des mf (10,0 mf/g). L’examen histologique des nodules collectés à 6 et 12 mois montrait que la FIAP n’était pas macrofilaricide mais avait un effet marqué sur l’embryogénèse. La nouvelle FIAP pourrait représenter un outil pour accélérer l’élimination de l’onchocercose dans certaines circonstances spécifiques.

## Introduction

The main control strategy for onchocerciasis is currently based on mass treatment with ivermectin (IVM) targeting the most affected populations, i.e. those (called meso-hyperendemic) where more than 20% of the adults present subcutaneous nodules containing *Onchocerca volvulus* adult worms. In Africa, community-directed treatment with IVM (CDTI) has led to the elimination of onchocerciasis as a public health problem in most of the treated areas. However, to reach the new World Health Organization’s objective of elimination of the infection [47], interventions might have to be expanded to the so-far untreated hypoendemic zones, and alternative treatment strategies (ATS, i.e., differing from annual CDTI) implemented [11]. Such strategies include the use of new drugs or new formulations of existing drugs.

IVM has two main effects on *O. volvulus*. First, it induces rapid destruction of the larval stage of the parasites (microfilariae [mf]) which are the cause of the immune reactions leading to the ocular and skin manifestations of the disease (microfilaricidal effect). Second, IVM treatment interrupts for 3–4 months the release of new mf by the adult female worms (embryostatic effect) [8]. However, as IVM has a limited effect on the lifespan of adult worms, CDTI has to be repeated every 6 or 12 months to maintain microfilarial densities (MFD) below the level associated with clinical manifestations. One of the ATSs that could be used to accelerate elimination of onchocerciasis would be to treat the whole population, or only those individuals currently infected with *O. volvulus*, with a macrofilaricidal drug, i.e. a drug that kills or permanently sterilizes the adult worms.

Presently, the only macrofilaricidal drug which can be distributed on a large scale without major risks of adverse effects is doxycycline. Daily treatment with this antibiotic for 4–6 weeks eliminates the *Wolbachia* symbiotic bacteria present in the adult worms, which leads to the sterilization and progressive death of the latter [45]. The main problem related to this strategy is the duration of the treatment, and research is ongoing to identify other drugs that could be macrofilaricidal using a regimen of two weeks or less. Three candidates have recently been tested as part of phase 1 trials. The first is oxfendazole [1], which belongs to the benzimidazole family and could have the advantage of killing the adult worms without affecting the mf, and thus would not induce adverse effects, particularly in case of coinfection with *Loa loa* [27, 48]. Ongoing trials (phase 1 and phase 2 against *Trichuris trichiura*) will enable us to evaluate its possible toxicity [25]. This point is key because the development of another benzimidazole to treat filariases, flubendazole, was interrupted in 2017 because its toxicity associated with effective doses was considered unacceptable [30]. The second macrofilaricidal candidate is emodepside, whose efficacy was demonstrated in pre-clinical trials [29], and which was evaluated in two phase 1 trials, one using single ascending doses [24], and the other using multiple ascending doses (https://clinicaltrials.gov/ct2/show/NCT03383614). The last candidate is a drug called TylAMac (Tylosin Analogue Macrofilaricide, ABBV-4083), which is a macrolide antibiotic effective against *Wolbachia* [41, 43], and which was also evaluated in a phase 1 trial terminated in 2018 (https://www.dndi.org/diseases-projects/portfolio/abbv-4083/). All these candidates are thus only at an initial stage of clinical development.

The present study was conducted following the observation that three-monthly doses of IVM induce excess mortality in adult worms, when compared to annual doses [22], this excess mortality actually being due to a significant decrease in the adult worms’ lifespan [46]. As the plasma half-life of IVM in man ranges between 12 h and 56 h following oral administration [26], we hypothesized that longer and continuous exposure of the parasite to the drug could have a stronger macrofilaricidal effect. Presently, several commercial long-acting formulations of IVM exist, one of the best known being the subcutaneous injectable formulation Ivomec^®^ Gold for cattle [15]. In this context, we tested the long-term efficacy (two years) of an injectable long-acting IVM formulation on the cattle-*Onchocerca ochengi* filarial model. This model is widely used to assess the effects of potentially filaricidal drugs because *O. ochengi* is taxonomically close to *O. volvulus*, and because the adult stages of both species live in subcutaneous nodules [32]. The tested formulation is based on the proprietary drug delivery platform BEPO^®^, which uses bioresorbable block copolymers as functional excipients to control the release of IVM. In this study, an assessment was made of the effect of the slow release of IVM on the skin MFD by counting mf in skin biopsies, and on the fertility and the viability of the adult worms, by histological examination of sections of excised subcutaneous nodules.

## Materials and methods

### Animals

Ten Gudali zebu cattle (*Bos indicus*) were purchased in villages near Ngaoundere (Adamaoua region of Cameroon) where transmission of *O. ochengi* is ongoing [44]. They were selected on the basis of their sex (female), age (three years) and presence of at least 10 subcutaneous nodules between the udder and the umbilicus, and in the inguinal region. The animals were not weighed, and no girth measurement was made to estimate their weight, but given the age of the cows, one can assume that weight ranged between 250 kg and 330 kg [2]. They were transported to the field research station of the Research Foundation for Tropical Diseases and the Environment (REFOTDE) located near Modeka, in the South–West region, on the right bank of the Mungo River. Each animal was identified using an individual number printed on labels attached at one ear. The interval of time between the departure of the cows from the Adamaoua region and the first examination round (and administration of treatment) was six weeks.

### Treatment description

The injectable long-acting formulation of IVM was prepared by Medincell using their proprietary drug delivery platform BEPO^®^ [23, 37]. A diblock (PEG–PLA) and a triblock (PLA–PEG–PLA) bioresorbable copolymer were allowed to dissolve overnight in a biocompatible solvent, dimethyl sulfoxide (DMSO), with gentle mixing on a roller mixer at room temperature. Then, 40 mL of the obtained vehicle were sterile filtered into a 50 mL glass bottle and kept refrigerated before shipment. The rest of the vehicle (approx. 160 mL) was used to prepare the IVM formulation; pre-weighed IVM powder was added to the vehicle and allowed to gently dissolve on a roller mixer at room temperature. The final solution of IVM was sterile filtered into a 250 mL glass bottle and kept refrigerated prior to shipment. The composition of the formulation was 7.5 w/w% IVM, 40 w/w% of copolymers and 52.5 w/w% of DMSO.

### Treatment and follow-up of adverse effects

Four animals (group A) were injected subcutaneously, just behind the shoulder, with 500 mg IVM, four others (group B) with 1000 mg IVM, and two others (group C) with the vehicle only. Injections were made by a veterinarian using a 16-gauge needle and the volume injected was about 5.8 mL for the 500 mg dose or 2 × 5.8 mL for the 1000 mg dose (5.8 mL behind each shoulder). As the animals’ weight was about 250–330 kg, the IVM doses administered in the two treated groups were 1.5–2.0 mg/kg and 3.0–4.0 mg/kg, respectively. Upon administration, the initially liquid formulation turned into a solid depot in the subcutaneous space, where IVM was released progressively by diffusion through the formed polymeric matrix. Any anomaly at the injection site(s) and relevant clinical signs (apathy, loss of appetite, tremors, locomotion problems, etc.) were monitored during the three days following the injection, and then at day 7.

### Pharmacokinetics

The blood sampling schedule was the following: in the vehicle group (group C): pre-dose (−1 h) and no subsequent sampling; in the IVM treated groups: pre-dose (−1 h) and multiple post-dose sampling (6 h, D2, D7, D14, D30, D90, D150, D180, D240, D300, D330, and D360).

At each time-point, atleast 4 mL of blood were withdrawn from the jugular vein or from a vein on the tail and transferred in ethylenediaminetetraacetic acid (EDTA) tubes to prevent coagulation. Samples were placed on ice before being centrifuged.

Blood collection tubes were promptly centrifuged at 2500 ×*g* for 10 min at room temperature and plasma was split in two aliquots of 500 μL in previously labelled polypropylene tubes (aliquots A and B (back-up sample)). Tubes with plasma specimens were frozen and stored at −80 °C until being shipped in dry ice containers to Europe. The samples were analyzed at the Echevarne Laboratory, Barcelona, Spain, using a liquid chromatography coupled with tandem mass spectrometry (LC/MS/MS) method. The bioanalytical method was validated based on the following criteria (Selectivity, Recovery, Carry-over, Calibration range and Response function, Limit of Quantification, Precision and Accuracy, Dilution Integrity, Matrix effect, Stability in samples, and Reference Solutions Stability). The Liquid Chromatography system was coupled to tandem mass spectrometry (Triple Quadrupole) with Electrospray Probe. Specifically, the chromatography was performed using a Synergi MAX204 RP column (100 Å, 100 × 3 mm, 2.5 μm) and a C18 guard cartridge (4 × 2 mm). The mobile phases were: A = 50 mM ammonium acetate (pH 4.5) and B = acetonitrile. The other conditions were: isocratic elution A/B (10:90); flow rate: 0.5 mL/min; injection volume: 10 μL; autosampler temperature: 4 °C; column temperature: 40 °C; and flush port: methanol. The detection consisted in multiple reaction monitoring (MRM) in positive mode. IVM was detected for m/z 892.400 > 307.200. The lower limit of quantification (LLOQ) for the method used was 0.1 ng of IVM per mL.

The analysis of the pharmacokinetic parameters (mean *C*_max_, *T*_max_, *C*_last_, and AUC_0–tlast_) was undertaken with the help of WinNonlin, Phoenix 64, version 8.0 software.

### Skin biopsies and nodulectomies

One skin biopsy was collected from each animal just before the subcutaneous injection (day 0, D0), and another one after 6, 12, and 24 months (M6, M12, and M24). Nodulectomies were performed at the same time as skin biopsies on D0, M6, and M12. To collect these samples, the animals were put in lateral recumbency, on a mattress, and maintained in this position with ropes, for a maximum of 1 h. All sample collections were performed by a veterinarian, after shaving of the skin and under local anaesthesia. Skin biopsies (surface area about 1 cm^2^) were taken from the area between the udder and the umbilicus, i.e. where the *O. ochengi* microfilarial densities are the highest [44]. Nodules were collected surgically. In some instances, more than one nodule was collected from a given animal at a given time-point. Nodules were placed in tubes containing 10 mL of fixative (10% formalin) until further processing. After nodulectomy, the wound was sutured and the animal received an intramuscular injection of antibiotics (streptomycin and penicillin G) with no action on the *Wolbachia* bacterial endosymbionts hosted by filariae. Stitches were removed after seven days.

### Microscopic examination of skin biopsies

The skin biopsies were left to incubate for 24 h at room temperature in 24-well plates, each well containing 300 μL of sterile RPMI 1640, and weighed with a 10 mg precision just before examination. The medium containing the mf which had emerged during the incubation period was pipetted and placed on microscopic slides and examined at ×40 and ×100 magnification. Four species of bovine *Onchocerca* are present in North Cameroon (*O. ochengi*, *O. gutturosa*, *O. dukei*, and *O. armillata* [44]), and mf were identified according to their size and aspect [6, 44]. No mf of *O. armillata* (length: 300–380 μm; width: 5.0–6.8 μm; characteristic bulging anterior end) was seen. Microfilariae of the three other species were observed: *O. ochengi* (length: 280–300 μm; width: 6–8 μm; rounded anterior end); *O. gutturosa* (length: 225–270 μm; width: 3.5–4.5 μm; rounded anterior end and tapering posterior end); and *O. dukei* (length: 220–260 μm; width: 5.0–6.5 μm; thinner anterior third of the body). Mf of each these three species were counted by two independent microscopists who had no information on the treatment received by the animal from which the biopsy was taken. The individual MFD were calculated as the arithmetic mean of the two counts and expressed as the number of mf per gram of skin. As mf of *O. dukei* were seen in only one animal at D0, with a low MFD (16.0/g), the results presented below distinguish only *O. ochengi* mf and “non *O. ochengi*” (i.e., *O. gutturosa* + *O. dukei*) mf. The MFD in each treatment group were calculated as the arithmetic means of individual MFD.

### Histological processing of nodules and assessment of worm viability and fertility

The nodules were embedded in paraffin wax, and 6 μm sections were stained with hematoxylin and eosin. Two of the authors (MB and SW) examined the sections independently and without having any information on the treatment arm of the animals. The nodules collected at each round of nodulectomies were examined separately. When the observers did not agree on the classification of the worms in a nodule, the slides were re-examined until a consensus was reached.

The reproductive status of the adult worms was assessed by the presence of oocytes and of embryos (morulas, coiled mf, and stretched mf). Live embryos were distinguished from degenerating ones [14]. Worms with uteri containing live embryos of any stage were considered fertile.

## Results

### Safety

After injection of the ~5.8 mL of liquid (5.8 mL × 2 for animals treated with 1000 mg IVM), a small bump (diameter: ~1 cm) could be palpated at the injection site. No side effects were recorded in any of the cows during the follow-up period.

### Pharmacokinetics

**Figure 1:**
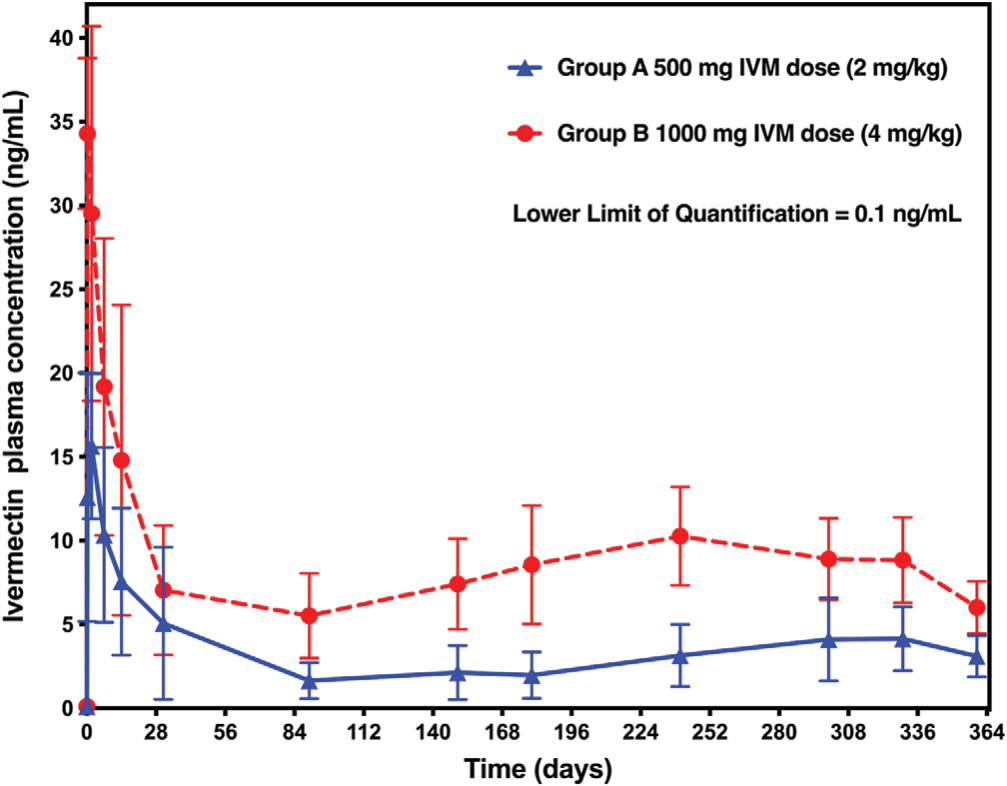
Plasma concentration-time profiles of IVM following subcutaneous injection of a long-acting formulation in cattle (*N* = 4 per group). LLOQ = lower limit of quantification.

Following the subcutaneous injection of the long-acting formulation of IVM in cattle, the obtained plasma-concentration time profiles were characterized by rapid absorption of the drug associated with a peak plasma concentration (*C*_max_) followed by sustained plasma concentrations for at least one year (Fig. 1). Mean *C*_max_, *T*_max_, *C*_last_ and AUC_0–tlast_ are presented in Table 1. As expected, there was a dose-dependent increase in the mean *C*_max_ and mean AUC_0–tlast_, the 3–4 mg/kg dose leading to a 2.2 times higher mean *C*_max_ and a 2.5 times higher mean AUC_0–tlast_ compared to the 1.5–2 mg/kg dose group. Consequently, steady IVM plasma concentrations were maintained in the range 5.52–10.28 ng/mL between days 90 and 365 for the 3–4 mg/kg dose and 1.65–4.15 ng/mL for the 1.5–2 mg/kg dose in the same period.

**Table 1:** Mean pharmacokinetic (PK) parameters in the two groups of animals treated with IVM.

PK parameters	Value	Treatment group	
		500 mg (1.5–2 mg/kg)	1000 mg (3–4 mg/kg)
*C*_max_ (ng/mL)	Mean	16.3	36.2
	SD	5.4	6.9
	CV (%)	33.2	19.1
*T*_max_ (days)	Median	2.00	0.25
*C*_last_ (ng/mL)	Mean	3.10	6.02
	SD	1.23	1.55
AUC_0–tlast_ (ng day/mL)	Mean	1239	3116
	SD	636	581

*C*_max_ = maximum observed plasma concentration; *T*_max_ = time of maximum observed plasma concentration; *C*_last_ = last measurable concentration (above the quantification limit); AUC_0–tlast_ = area under the concentration-time curve to the last measurable concentration; SD = standard deviation; CV = coefficient of variation.

### Microfilarial densities

The individual and mean MFDs for *O. ochengi* are shown in Table 2 and those for non-*ochengi Onchocerca* are presented in Table 3. At D0, one of the cows of group B (cow number 3) did not present *O. ochengi* mf. After excluding this animal, the mean *O. ochengi* MFDs were 367.0 mf/g in the nine remaining cows (standard deviation [SD] = 398.6), and 471.6 (SD = 540.1), 296.5 (SD = 188.2), and 263.8 (SD = 196.2) mf/g in cows of groups A, B, and C, respectively. The pre-treatment non-*ochengi* MFDs were 332.3 (SD = 386.9), 230.9 (SD = 193.9), and 17.3 (SD = 2.6) in groups A, B, and C, respectively.

**Table 2:** *Onchocerca ochengi* MFD per gram of skin in each animal and each treatment group at D0, M6, M12, and M24. Post-treatment-positive results are shown in bold.

Group^a^	ID	D0			M6			M12			M24		
		W	No. Oo	MFD Oo	W	No. Oo	MFD Oo	W	No. Oo	MFD Oo	W	No. Oo	MFD Oo
A	4	0.73	15	20.5	0.18	0	0	1.90	0	0	0.92	0	0
A	9	0.34	177	520.6	0.31	0	0	1.00	0	0	0.70	**7**	**10.0**
A	28	0.25	334	1336.0	0.24	0	0	1.10	**1**	**0.9**	0.87	0	0
A	294	0.22	2	9.1	0.99	0	0	1.30	0	0	1.47	0	0
	Mean^b^			471.6			0			0.2			2.5
B	3	0.30	0	0	0.44	0	0	1.10	0	0	0.32	0	0
B	92	0.18	6	33.3	0.30	0	0	1.10	0	0	0.86	0	0
B	147	0.62	287	462.9	0.47	0	0	0.30	0	0	1.36	0	0
B	255	0.44	173	393.2	0.16	0	0	1.50	0	0	0.13	0	0
	Mean^b^			296.5			0			0			0
C	254	0.45	207	460.0	0.32	**120**	**375.0**	0.90	**1110**	**1233.3**	0.52	**120**	**230.8**
C	256	0.37	25	67.6	0.50	**137**	**274.0**	0.40	**187**	**467.5**	0.52	**1335**	**2567.3**
	Mean^b^			263.8			324.5			850.4			1399.0

*Abbreviations*: ID = animal identity number; W = weight of skin specimen (in grams); No. Oo = total number of microfilariae of *Onchocerca ochengi* having emerged from the skin specimen; MFD Oo = *O. ochengi* microfilarial density per gram of skin.

a A = 500 mg IVM; B = 1000 mg IVM; C = Control (vehicle only).

b Arithmetic mean of the individual *Onchocerca ochengi* microfilarial densities in the group (in group B, the mean was calculated after excluding animal #3, which did not present *O. ochengi* microfilariae before treatment).

**Table 3:** Non-*O. ochengi* (*O. gutturosa* and *O. dukei*) MFD per gram of skin in each animal and each treatment group at D0, M6, M12, and M24. Post-treatment-positive results are shown in bold.

Group^a^	ID	D0			M6			M12			M24		
		W	No. Ogd	MFD Ogd	W	No. Ogd	MFD Ogd	W	No. Ogd	MFD Ogd	W	No. Ogd	MFD Ogd
A	4	0.73	2	2.7	0.18	0	0	1.90	0	0	0.92	0	0
A	9	0.34	90	264.7	0.31	0	0	1.00	0	0	0.70	0	0
A	28	0.25	20	80.0	0.24	0	0	1.10	0	0	0.87	0	0
A	294	0.22	216	981.8	0.99	0	0	1.30	0	0	1.47	0	0
	Mean^b^			332.3			0			0			0
B	3	0.30	11	36.7	0.44	0	0	1.10	0	0	0.32	0	0
B	92	0.18	92	511.1	0.30	0	0	1.10	0	0	0.86	0	0
B	147	0.62	40	64.5	0.47	0	0	0.30	0	0	1.36	0	0
B	255	0.44	137	311.4	0.16	0	0	1.50	0	0	0.13	0	0
	Mean^b^			230.9			0			0			0
C	254	0.45	9	20.0	0.32	**21**	**65.6**	0.90	0	0	0.52	**59**	**113.5**
C	256	0.37	5	14.7	0.50	**7**	**14.0**	0.40	**27**	**67.5**	0.52	0	0
	Mean^b^			17.3			39.8			33.7			56.7

*Abbreviations*: ID = animal identity number; W = weight of skin specimen (in grams); No. Ogd = total number of microfilariae of *Onchocerca gutturosa* or *O. dukei* having emerged from the skin specimen; MFD Ogd = *O. gutturosa* + *O. dukei* microfilarial density per gram of skin.

a A = 500 mg IVM; B = 1000 mg IVM; C = Control (vehicle only).

b Arithmetic mean of the individual *Onchocerca ochengi* microfilarial densities in the group.

The *O. ochengi* mean MFD increased gradually from D0 to M24 in animals in group C: 324.5, 850.4, and 1399.0 mf/g at M6, M12, and M24 (SD = 50.5, 532.9, and 1168.3), respectively. The non-*ochengi* MFDs in this control group fluctuated between 33.7 mf/g and 56.7 mf/g.

At M6, no mf (*O. ochengi* or non-*ochengi*) was found in the skin samples taken from the cows treated with IVM. At M12, only one animal treated with IVM was found positive for *O. ochengi*, with only one mf seen in the incubation liquid (MFD: 0.9 mf/g). The cow was the one that had the highest pre-treatment MFD and belonged to group A (thus treated with the “low” dose of IVM). At M24, none of the cows treated with IVM showed non-*ochengi* skin mf but, again, one cow in group A showed *O. ochengi* mf (MFD: 10.0 mf/g). This cow was the one that had the second highest pre-treatment MFD: 520.6 mf/g. The cow found positive at M12 was negative at M24.

### Reproductive status and viability of adult worms

Sixteen nodules containing 24 female worms were collected on D0, just before treatment. Six of the females (25%) contained live embryos in their uteri and were thus considered fertile, and 19 (79.2%) contained live embryos or oocytes and were thus fertile or potentially fertile (results in Table 4). Similar percentages (29.0 and 74.2%, respectively) were obtained by combining all the untreated female worms (thus including the worms collected at M6 and M12 from animals in group C).

**Table 4:** Results of the histological examination of nodules.

Time point	Treatment group(s)^a^	No. nodules	No. female worms	No. fertile	No. shedding oocytes	No. empty	No. dead	% fertile females	% worms fertile or shedding oocytes
D0	A	6	12	1	8	2	1	8.3	75.0
	B	6	7	2	3	1	1	28.6	71.4
	C	5	5	3	2	0	0	60.0	100
	A + B + C	16	24	6	13	3	2	25.0	79.2
M6	A	7	6	0	5	1	0	0	83.3
	B	5	5	0	5	0	0	0	100
	C	3	1	1	0	0	0	100	100
M12	A	8	8	0	3	5	0	0	37.5
	B	7	5	0	2	3	0	0	40.0
	C	5	6	2	1	1	2	33.3	50.0
M6	A + B	12	11	0	10	1	0	0	90.9
M12	A + B	15	13	0	5	8	0	0	38.5
All naïve worms^b^		24	31	9	14	4	4	29.0	74.2

a A = 500 mg IVM; B = 1000 mg IVM; C = control (vehicle only).

b All worms (groups A, B, and C) collected at D0 and worms in group C collected at M6 and M12.

On M6 and M12, none of the female worms collected from the treated groups (*N* = 11 on M6 and 13 on M12) contained live embryos in their uteri. The proportion of potentially fertile females (shedding oocytes), which was fairly high on M6 in the treated groups (90.9%), decreased to 38.5% at M12.

During the course of this study, no dead female worms were observed in the treated groups of cows at M6 and M12 (total number of females observed at these time-points: *N* = 11 and *N* = 13, respectively). Conversely, 4 of the 31 untreated female worms (worms observed at D0 in nodules collected from the three groups, plus worms observed at M6 and M12 in nodules from group C) were dead (12.9%). These results suggest that the subcutaneous injectable long-acting formulation of IVM did not have a detectable macrofilaricidal effect on the adult worms.

Male worms were not counted because their numbers were very low in the examined histologic sections. In addition, some sections were of sub-optimal quality and enabled only assessment of female worms.

## Discussion

Wahl et al. [44] assessed the anatomic distribution of *O. ochengi* mf in the hide of eight cows infected with this parasite. At the sites of highest concentration (near the umbilicus and in the inguinal region) the MFD recorded after 4-h incubation in RPMI was 221 mf/g. As the MFD increases by 1.5–2.0 when the incubation time increases from 4 h to 24 h [44], this MFD was similar to that recorded during the present study (367.0 mf/g).

Our results show that the *in-situ* forming depot containing IVM used in this study maintained the *Onchocerca* sp. mf at extremely low levels for two years. None of the cows treated with IVM presented skin mf at M6, and only one was mf-positive at M12 (with 0.9 mf/g), and another at M24 (with 10.0 mf/g). Both the cows with post-treatment-positive biopsies had been treated with the low dose (500 mg) of IVM. These results were obtained by examining a single fairly large biopsy (180–730 mg), and not smaller biopsies (mean weight: 54 mg) taken from three different sites, as done in another study [36]. By doing so (mainly to limit the time the animals were held down in an uncomfortable position), we could not account for variation in MFD in the skin [44], but given the considerable decrease in the MFD observed in the IVM-treated animals, the results would have probably been similar by examining more than one biopsy.

Interestingly, in cows in group C, the MFD increased gradually from D0 to M24, both for *O. ochengi* (from 264 mf/g to 1399 mf/g) and non-*O. ochengi* (from 17.3 mf/g to 56.7 mf/g). As the REFOTDE field research station is located in an area where there is probably no transmission of bovine *Onchocerca* spp., this increase might be due to the fact that pre-adult or young adult stages which were present at D0 developed during the following two years to adult worms producing mf. This increase in the MFD in group C makes the persistent absence of mf for two years in most of the cows in groups A and B even more remarkable.

The possibility that the decrease in the MFD could be due, at last partly, to other treatments received by the animals before their departure from the Adamaoua region has to be considered. Even though little quantitative information is available on the veterinary drug market in this area, it is known that levamisole, albendazole and IVM are widely used by cattle herders to treat intestinal nematode infections in their animals [20]. Levamisole has no significant effect on the microfilariae and macrofilariae of *O. volvulus* [3], and this is probably also the case for *O. ochengi*. A single dose of albendazole (400 mg) has little effect on the *O. volvulus* MFD and adult-worm reproductive activity [5], but treatment with 800 mg daily for three or seven days, or with 1200 mg daily for three days leads to a gradual decrease in the MFDs, which are reduced by 24–66% one year after treatment [4]; the latter regimens have no macrofilaricidal effect, and the effect on the MFD is due to an embryotoxic effect (i.e., preventing the embryos from developing in the uteri to the stretched mf stage). Regarding IVM, it is known that *O. volvulus* MFDs are reduced by 99% of pre-treatment levels 1–2 months after treatment, and then re-increase progressively [8]. As the dynamics are probably similar for *O. ochengi* [36], if the drug had been given just before the animals’ departure from the Adamaoua region, then the *O. ochengi* MFD would have been close to their nadir when the cows underwent pre-treatment biopsy six weeks later. As most of the study animals showed significant *O. ochengi* or non-*ochengi* MFD at that time, we can assume that they had not been treated with IVM recently; and should this be the case, then such treatment could not explain the subsequent decrease in the MFD. These considerations, together with the fact that the substantial decrease in the MFD was seen only in those cows that received IVM, lead us to believe that any treatment received by the animals before the study had only a minimal influence, if any, on the results observed.

The prolonged effect of the formulation on the MFD could be due to an embryotoxic (see above) and/or an embryostatic effect (preventing the release of mf from the adult female worms), and/or a persisting microfilaricidal effect (destruction of the mf after their release from the females’ uteri), and/or a macrofilaricidal effect (killing of the adult worms). As the main objective of the study was to investigate the effects of the formulation on the adult worms, we did not collect skin samples within the first weeks following treatment to evaluate the (probable) microfilaricidal effect. The histologic examination of nodules collected at M6 and M12 provided information on the effects of the formulation on the adult worms’ viability and reproductive status. This showed that all the female worms collected at M6 and M12 from the cows treated with IVM were alive, showing that the formulation had no macrofilaricidal effect. These results are similar to those obtained, also using the bovine-*O. ochengi* model, with repeated monthly doses of subcutaneous IVM at 500 μg/kg [12]. However, the females from animals treated with IVM did not contain live embryos in their uteri, demonstrating that the treatment had a strong effect on the parasite’s fertility. In addition, the fact that the proportion of worms shedding oocytes decreased markedly between M6 and M12 suggests that permanent exposure to IVM for one year might sterilize the worms. No nodules were collected at M24, but the fact that one (and only one) cow treated with IVM showed skin mf at that time-point suggests that the condition of the worms at M24 was probably similar to that observed at M12.

Given these results, one may wonder what role the *in-situ* forming depot evaluated as part of this study could play to accelerate the elimination of human onchocerciasis, and thus whether it would be worth assessing this formulation in phase 1 clinical trials. The long-term effect of the IVM-releasing depot on *O. ochengi* MFD was remarkable and a comparable effect would probably be obtained with *O. volvulus*. However, a similar effect on the MFD, and thus on the transmission of *O. volvulus*, could be obtained with annual oral treatment with moxidectin (MOX), a drug whose plasma half-life is 20–43 days, i.e. much longer than that of IVM (12–56 h) [35]. The question to be answered is “what would be the advantage of treating with an injectable long-acting formulation of IVM, instead of annual oral doses of MOX?” As IVM seems to have a prophylactic effect on *Onchocerca* sp. [40, 42], i.e. prevents the development of the parasite up to the adult stage, a sustained-release formulation might protect people from new infections for many months. A solid implant containing IVM was shown to be effective in preventing experimental infection of dogs with *Dirofilaria immitis* larvae [18]. The prophylactic effect of MOX on *Onchocerca* sp. is unknown. Injectable long-acting formulations of MOX (ProHeart 6 and ProHeart 12) are used to prevent canine infection with *D. immitis* [34] but, given the half-life of the drug, oral treatment with MOX would probably need to be repeated every 2–3 months to have any prophylactic effect against *O. volvulus*. In this case, an IVM-containing subcutaneous depot might be advantageous. The prophylactic effect of an injectable long-acting formulation of IVM on *O. ochengi* could be tested using the same protocol as that used for IVM [42], i.e. by comparing the incidence of infection in two groups of calves living in an area where the parasite is transmitted, one treated with the IVM formulation, and one receiving only the vehicle.

In addition, macrocyclic lactones like IVM and MOX are also effective against soil-transmitted helminths (STH) and ectoparasites such as scabies and lice [7]. A yearly single subcutaneous injection of a long-acting formulation of IVM might be as efficient to prevent clinical manifestations of STH as two- or three-monthly doses of MOX. Regarding scabies, *in vitro* assays suggest that the concentration of MOX required to kill the mites might be lower than that of IVM [33], and trials using a porcine model suggest that a single oral dose of MOX is more effective than two consecutive IVM doses [10]. Collateral benefits of a sustained-release formulation of IVM would also include an effect on the longevity of mosquitoes and other insects biting treated people. Studies are being conducted to develop long-acting formulations of IVM that could help decrease the density of *Anopheles* sp. and thus the transmission of malaria, and an oral ultra-long-acting drug delivery system containing IVM was developed to reach this objective [9]. A subcutaneous IVM-releasing depot could have the same effect, with the significant advantage of showing sustained release over up to a year. In addition, when the malaria vectors are zoophagic, treatment of cattle could play a significant role toward reducing vector density, and thus malaria transmission [16]. Conversely, repeated doses of MOX might well have little impact on malaria transmission because MOX seems to be less active than IVM on malaria vectors [13, 21].

The fact that the long-acting formulation of IVM used as part of this study does not seem to be macrofilaricidal for *O. volvulus* is disappointing. However, it seems to sterilize the female worms, and this, added to other effects against soil-transmitted helminths and some ectoparasites and vectors of serious diseases like malaria, makes the concept of a long-acting formulation of IVM a potentially highly valuable alternative to the existing methods, including the use of oral IVM (and/or MOX) tablets.

Of course, any decision regarding treatment with long-acting formulations has to be taken considering possible risks associated with long-term exposure to these drugs. For IVM long-acting formulations, one of the risks is the possible accelerated selection of IVM-resistant parasites, including *Onchocerca* sp. or intestinal nematodes [39]. Studies have suggested that the embryostatic effect of IVM against *O. volvulus* could be reduced in populations treated repeatedly with IVM, but this phenomenon might not be due to genetic selection, but to other processes that remain to be clarified [19]. Regarding intestinal nematodes, the risk of resistance is certainly higher than for filariae, but it could be prevented by treating the host simultaneously with another anthelmintic such as a benzimidazole. The second point to consider is the management of drug-drug interactions (DDIs) in subjects already treated with another drug, or who have to start treatment with another drug after injection of the IVM long-acting formulation. A review of the interactions of IVM (and other macrocyclic lactones) with ATP-binding cassette transporters suggests that co-administration of IVM with drugs such as the antifungal drug ketoconazole, the antihypertensive, and antiarrhythmic drug verapamil, or the anti-diarrheal drug loperamide can increase the IVM AUC by 2-fold [31]. In addition, *in vitro* or animal model studies suggest possible DDIs between IVM and antibiotics or antiretroviral drugs, and further investigations should be conducted to investigate the possibility of *in vivo* interactions in humans [28]. This being said, one must recall here that the depot formed by the IVM-long acting formulation used as part of this study can be easily removed if necessary, to prevent adverse effects due to DDIs.

Presently, the major indications for subcutaneous implantable devices or injectable long-acting formulations in humans include contraception (implants containing levonorgestrel or etonogestrel or *in-situ* formed depots containing medroxyprogesterone acetate (MPA)), treatment of schizophrenia (*in-situ* formed depot containing risperidone), treatment of prostate cancer (implants or depot containing goserelin, leuprolide, or histrelin), and treatment of opioid abuse (*in-situ* formed depot containing buprenorphine) [38]. Sayana^®^ Press, a formulation containing 104 mg MPA in a 0.65 mL suspension and which can be injected subcutaneously by trained community health workers or self-injected, is a very popular family planning method in Africa [17]. More than one million doses have been used so far. It would certainly be useful to conduct socio-anthropologic studies in population where onchocerciasis and malaria are endemic to evaluate the acceptability of a subcutaneous injection of a long-acting formulation of IVM that is fully bioresorbable and would therefore not require depot removal upon completion of the release period.

## Conflicts of interest

The patent related to the formulation used during this study belongs to MedinCell S.A. There is no conflict of interest between the co-authors and present or past affiliation with MedinCell and the co-authors affiliated at the *Institut de Recherche pour le Développement* (IRD) and the Research Foundation for Tropical Diseases and the Environment (REFODTE). Co-authors affiliated with IRD or REFODTE have no specific interest (i.e., shares) or commercial relationship (i.e., consulting) with MedinCell. In the event of a commercial development of the long-acting formulation of IVM described in the present publication, MedinCell would benefit from the outcomes of the present study. However, the co-authors with present or past affiliation at MedinCell did not contribute to the examination of the skin samples or the onchocercal nodules collected as part of this study, nor to the data analysis and interpretation of results.
